# Membrane traffic research: challenges for the next decade

**DOI:** 10.3389/fcell.2014.00052

**Published:** 2014-09-17

**Authors:** Gerard Apodaca, William J. Brown

**Affiliations:** ^1^The Renal-Electrolyte Division, Department of Medicine, University of PittsburghPittsburgh, PA, USA; ^2^Molecular Biology and Genetics, Cornell UniversityIthaca, NY, USA

**Keywords:** membrane contact sites, mTORC1 signaling, SREBPs, lipid droplets, store-operated Ca2+ entry, Notch signaling, umbrella cell, t-cell synapse

## Introduction

The study of membrane traffic is now a well-established area of research, and one that has resulted in several Nobel prizes including ones awarded to Albert Claude, George Palade, and Christian DeDuve in 1974, Michael Brown and Joseph Goldstein in 1985, Gunter Blobel in 1999, and most recently James Rothman, Randy Schekman, and Thomas Südhof in 2013. As a result of their studies and other research, we now have fundamental insights into the organization and routes of transport between the cells' membranous organelles. Moreover, we have defined the basic “cellular machinery” that governs protein and lipid synthesis, that ensures selective recognition of proteins and lipids, and that promotes vesicle fission, transport, and fusion. In addition, we have a large number of insights into the regulatory molecules that control these processes including the Rab GTPases and their effectors. While the challenges for the future are many, this essay is focused on areas of investigation that we see as moving forward at a rapid pace, which speak to how membrane traffic contributes to overall cell and tissue function, and which are likely to provide important avenues of funding for both established and new investigators. These challenges include how membrane traffic is regulated in response to metabolic needs, how molecules are transferred between organelles, how membrane traffic is regulated and functions during processes such as development, and how membrane traffic is used by highly differentiated cells to perform specialized cell functions.

## How is membrane traffic regulated in response to metabolic needs?

Membrane traffic is known to be regulated by extracellular cues including growth factors and neurotransmitters; however, significantly less is known about how the cell modulates its membrane traffic to match its metabolic needs. This is critical during processes such as cell growth, where membranes must be expanded in advance of cell division. Moreover, many diseases that affect large swaths of our population, including diabetes and obesity, are essentially metabolic disorders. Considering the primary role that cells play in anabolism and catabolism, understanding how these processes are coordinated with membrane traffic is a challenge that is very likely to enjoy enhanced attention and future research.

### mTORC1, lysosome biogenesis, and autophagy

**Figure 1:**
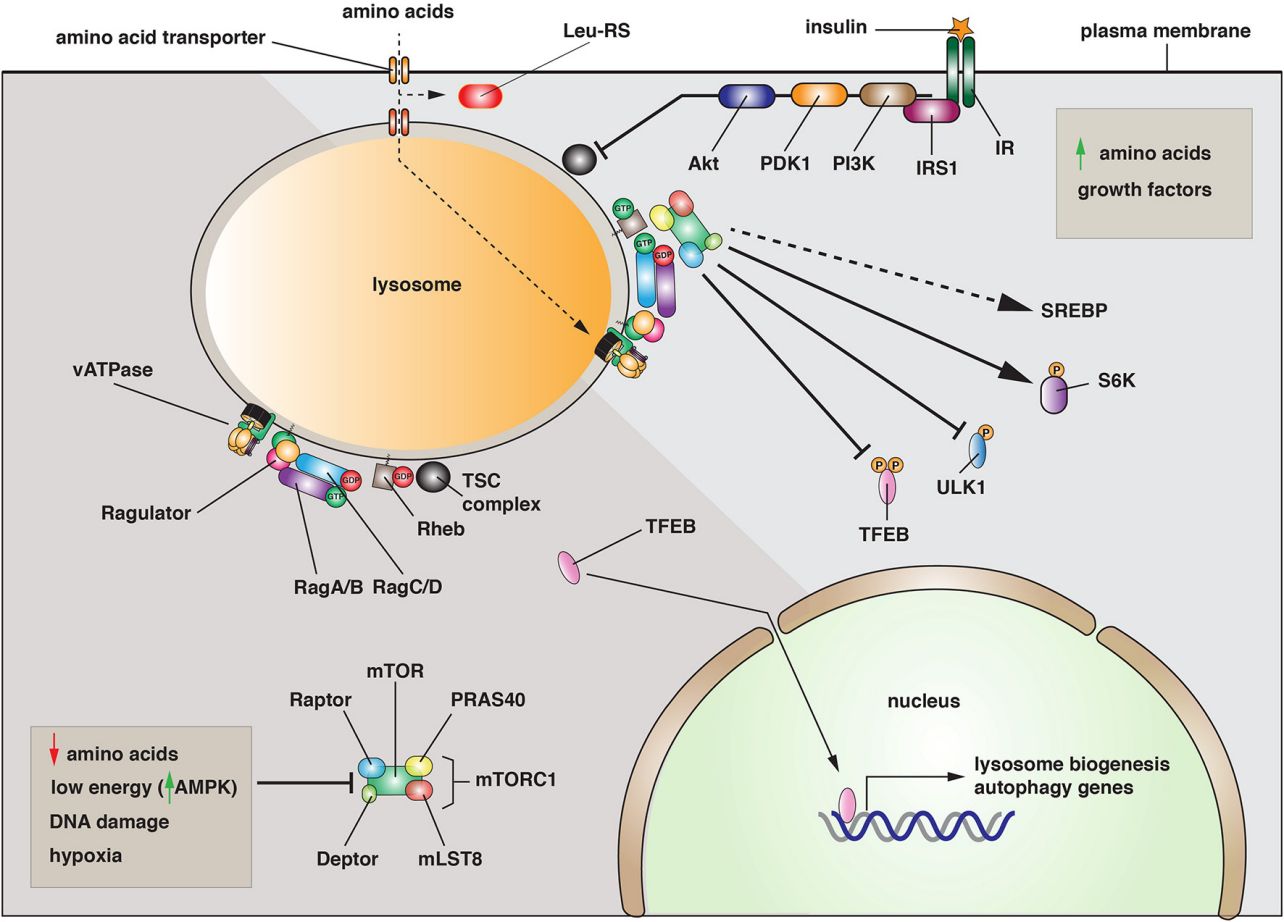
**Activation and function of the mTORC1 complex. Left:** Under conditions when amino acids are depleted, when energy levels are low, or when DNA is damaged, the mTORC1 complex is inactive and primarily cytosolic. Under these conditions, the transcription factor TFEB enters the nucleus where it promotes the expression of genes necessary for lysosome biogenesis and autophagy. **Right:** When amino acids are elevated, the mTORC1 complex is recruited to the lysosomal membrane by the combined effects of the Rag and Rheb GTPases. In its activated state, mTOR phosphorylates several downstream targets including TFEB, the autophagy regulatory factor ULK1, and the S6-kinases (S6K). Phosphorylation of TFEB or ULK1 is inhibitory, while phosphorylation of S6Ks leads to increased protein synthesis. mTORC1 is also activated by growth factors such as the insulin receptor (IR). Upon insulin binding, a signaling cascade is initiated by the insulin receptor substrate 1 (IRS1) and continues with recruitment of phosphoinositide-3-kinase (PI3K), the 3-phosphoinositide dependent protein kinase-1 (PDK1), and the Akt kinase. The latter inhibits the activity of the tuberosclerosis complex (TSC), leading to Rheb activation.

An important regulatory pathway is the mechanistic (previously mammalian) target of rapamycin (mTOR), a serine/threonine kinase at the heart of mTOR complex-1 (mTORC1) (Betz and Hall, 2013). This protein assembly also includes Raptor, mLST8, Deptor, and PRAS40 (Jewell et al., 2013). mTORC1 plays a critical role in integrating growth control by sensing the nutrient and energy status of the cell along with the presence of growth factors. When the cell is energy replete, and amino acids are present, mTORC1 is recruited to lysosomal membranes by the activity of the Ragulator (composed of p14, p18, MP1, C7orf59, and HBXIP) (Bar-Peled et al., 2012;Betz and Hall, 2013) (Figure 1). The Ragulator functions as a guanine nucleotide exchange factor (GEF) for the GTPases RagA/B, which act in concert with RagC/D to recruit mTORC1 to the lysosomal membrane (Kim et al., 2008; Sancak et al., 2008). Rag GTPase-activating proteins (GAPs) have not been identified and it is unknown if Rags cycle on and off the lysosomal membrane. Once recruited, and in a reaction that depends on the Rheb GTPase, mTOR is activated and stimulates ribosome biogenesis and translation by phosphorylating the ribosomal S6 kinases, and lipid synthesis (Figure 1). Rheb activity is regulated by the tuberous sclerosis (TSC) complex, which includes TSC1, TSC2, and TBC1D7. Collectively, this complex forms a Rheb-specific GAP that is negatively regulated by growth factors that act through Akt (Manning and Cantley, 2007). Whereas mTORC1 stimulates biosynthetic pathways, activated mTORC1 negatively regulates lysosome biogenesis and autophagy by phosphorylating and preventing the nuclear translocation of TFEB, a transcription factor that regulates expression of genes that control lysosome biogenesis and autophagy (Martina et al., 2012; Roczniak-Ferguson et al., 2012) (Figure 1).

Why recruit mTORC1 to the lysosome? Studies to date indicate that this may be a critical mechanism to couple mTORC1 activation to amino acid availability (Efeyan et al., 2012). In yeast, there is significant evidence that the vacuole is a storage site for amino acids, and mammalian lysosomes may perform a similar function. Intriguingly, addition of amino acids to purified lysosomes is sufficient to recruit mTORC1 to the organellar membrane (Zoncu et al., 2011). These and other data indicate that there is a mechanism for amino acid uptake into the lysosome that controls Rag activation by an inside-out mechanism that conveys information about amino acid levels in the lysosome lumen to the Rags and Ragulator (Zoncu et al., 2011; Efeyan et al., 2012). Interestingly, the vacuolar ATPase plays an important but poorly understood role in this process (Jewell et al., 2013). One model is that amino acid transporter function is linked to the proton gradients formed by the vacuolar (v)-ATPase (i.e., symport or antiport mechanisms). However, an alternative model is that the v-ATPase functions by maintaining the pH of the lysosome and/or the pH of the cytoplasm. In either case, there is limited understanding of how amino acids are transported into the lysosome or how this information is transferred in a v-ATPase dependent manner to mTORC1 recruitment. To add to the complexity, there may be a mechanism for cytoplasmic sensing of leucine, which involves the leucyl-tRNA synthetase and RagD (Bonfils et al., 2012; Han et al., 2012). But how this interaction is linked to mTORC1 localization and function remains to be determined.

When amino acids fall below a poorly defined set point, mTORC1 becomes inhibited and is released from the lysosome into the cytosol. As a result, TFEB is no longer phosphorylated by mTORC1. In its dephosphorylated state, TFEB is translocated into the nucleus where it binds to the “coordinated lysosomal expression and regulation” (CLEAR) promoter elements that control expression of several lysosomal and autophagy-related genes (Palmieri et al., 2011; Roczniak-Ferguson et al., 2012) (Figure 1). Combined with dephosphorylation and activation of the autophagy-inducing kinase ULK1, there is massive formation of autophagosomes, which undergo fusion with lysosomes to create a hybrid organelle that promotes degradation. In effect, the cell increases the pool of available amino acids by stimulating protein turnover. Intriguingly, mTORC1 is subsequently recruited to the autophagolyososomes where it now stimulates the re-formation of lysosomes that bud from the hybrid organelle (Yu et al., 2010; Efeyan et al., 2012). What mTORC1 does to potentiate this fission reaction is poorly understood. While growth factors are an additional mechanism for stimulating mTORC1 activation/lysosome recruitment, inhibitory factors such as decreased energy levels, which activate the AMP-regulated kinase (AMPK), DNA damage, or hypoxia can all act to prevent mTORC1 activation and recruitment to the lysosome (Efeyan et al., 2012) (Figure 1). Thus, a number of stressful stimuli can target mTORC1-regulated pathways such as lysosome biogenesis and autophagy.

In addition to mTORC1, there is an additional related complex called mTORC2 (Betz and Hall, 2013). It is comprised of mTOR, Rictor, SIN1, and mLST8. This complex is known to regulate lipogenesis, glucose metabolism (by way of the classical Akt pathway), the actin cytoskeleton, and apoptosis. Whereas mTORC1 is found prominently at the lysosome, mTORC2 is located at the endoplasmic reticulum (ER) and mitochondria, and perhaps at the closely apposed contact sites between mitochondria and ER membranes (Betz and Hall, 2013). These so-called “membrane contact sites” (MCSs) are described in more detail below. What mTORC2 does at these sites is unknown.

In summary, mTORC1/2 complexes appear to play a critical role in matching membrane traffic to intrinsic and extrinsic cues such as cellular ATP and amino acid levels, and the presence of hormones (e.g., insulin) and growth factors (e.g., EGF receptor ligands and insulin-like growth factor 1). In autophagy and lysosome biogenesis, there is a growing understanding of how mTORC1 associates with the relevant membranes and how it upregulates these pathways upon nutrient depletion. However, how or if mTORC1/2 regulates other membrane trafficking pathways is largely unexplored, even though mTORC1 and/or MTORC2 are found at the plasma membrane, mitochondria and peroxisomes, as well as the nucleus (Betz and Hall, 2013). Thus, an important future challenge is to understand how mTORC complexes are selectively recruited to membranes other than lysosomes, to define the effectors of mTORC1/2 at these disparate membrane domains, and to establish how these effectors stimulate/repress membrane trafficking events.

### SREBP and lipid metabolism

**Figure 2:**
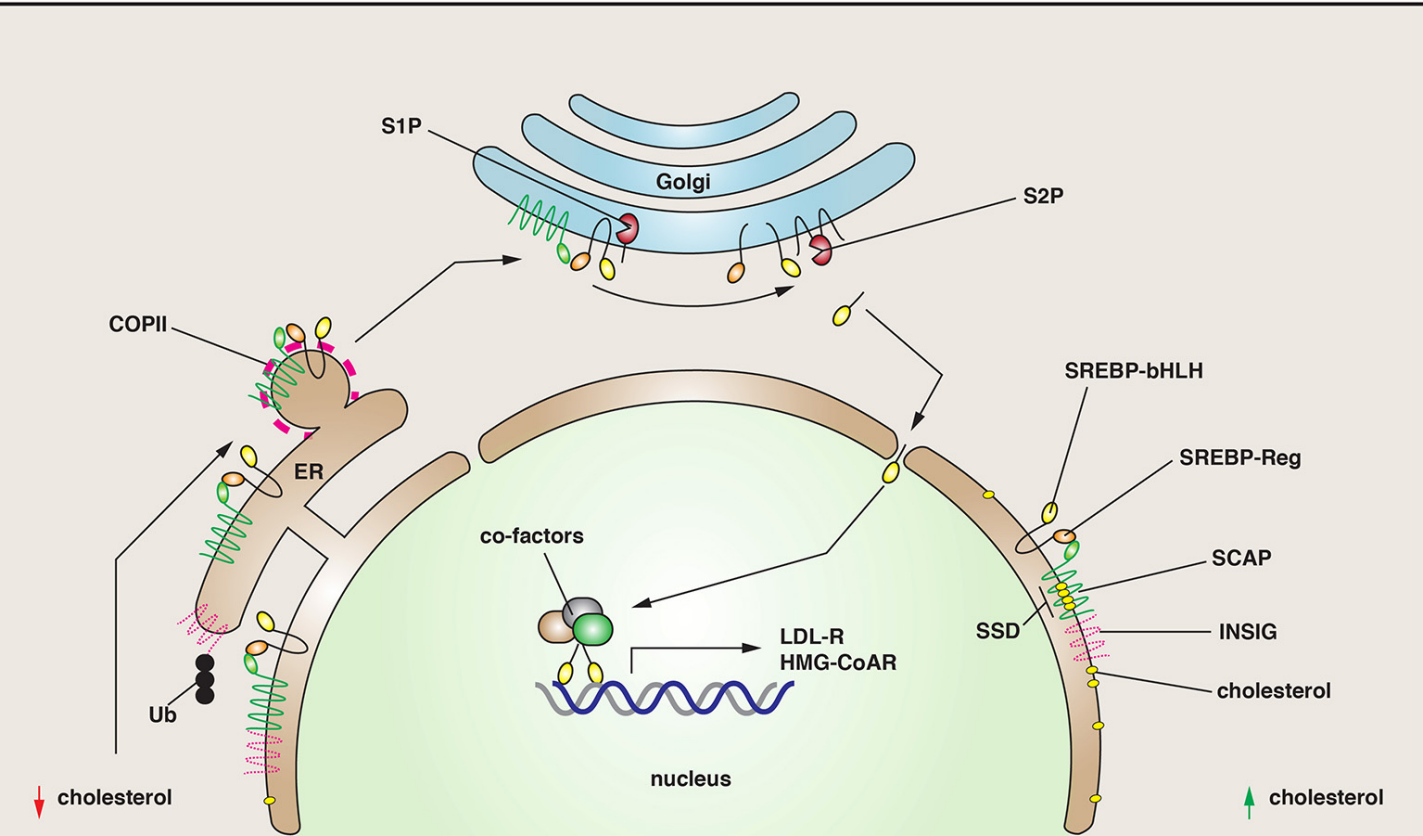
**SREBP cleavage and function**. SREBP is a regulator of genes involved in lipid metabolism. It has two critical domains: a regulatory one (SREBP-Reg) that promotes interactions with SCAP, and one with a basic helix-loop-helix structure (SREBP-bHLH), a structure characteristic of transcription factors. **Right:** Under conditions where cholesterol levels are elevated, SREBP is maintained in the ER via its interactions with SCAP, a protein that has a sterol-sensing domain (SSD). SCAP is maintained in the ER through inhibitory interactions with INSIG. **Left:** As cholesterol levels fall, SCAP senses the change, disengages from INSIG, which is ubiquitinated (Ub) and degraded. The SCAP/SREBP complex is then packaged into COPII-coated vesicles. In the Golgi, SREBP is cleaved first by the site-1-proteinase (S1P), and then the site-2-proteinase (S2P), releasing the bHLH domain. This domain enters the nucleus where it stimulates the transcription of genes involved in sterol biogenesis.

Understanding lipid metabolism is important because lipids and sterols are critical precursors of steroid hormones, they are the basis of several critical signaling pathways in the cell, and they are key components of cellular membranes. One mechanism for sensing changes in cholesterol levels requires the “sterol regulatory element binding proteins” (SREBPs), three subtypes of which have been identified (Daemen et al., 2013). They are nominally ER resident proteins that are cleaved by intramembrane proteolysis in response to depletion of lipids and sterols (Wang et al., 1994) (Figure 2). The released N-terminal domain acts as a transcription factor to stimulate lipid and sterol biosynthesis by increasing gene expression of proteins involved in cholesterol biogenesis (Hua et al., 1993; Yokoyama et al., 1993; Wang et al., 1994). One target of this transcription regulation is the LDL receptor, a classical recycling receptor that scavenges lipid-rich particles in the blood (Brown and Goldstein, 1997). Additional targets include enzymes such as the HMG-COA reductase, which catalyzes the rate-limiting step in cholesterol biogenesis. The function of SREBP depends on its association with the “SREBP cleavage-activating protein” (SCAP) (Figure 2), which has an N-terminal sterol-sensing domain found in other proteins including the HMG-COA reductase, the Niemann Pick disease type C1 protein, and Patched, a critical component of the Hippo pathway (Hua et al., 1996; Daemen et al., 2013). In response to sterol depletion, SCAP is thought to change its conformation, releasing it from the effects of an inhibitory protein called the “insulin-induced gene” (Insig) (Sever et al., 2003). Active SCAP then interacts with SREBP, promoting COPII-dependent transport of SCAP-SREBP from the ER to the Golgi complex, where SREBP is cleaved by SP2 (Figure 2).

There is a growing realization that the SREBP pathway is not just regulated by lipids/sterols, but also by glucose, insulin, and other metabolic intermediates (Daemen et al., 2013). In the case of insulin, the PI3K/Akt pathway may play a critical role by regulating the traffic of the SCAP-SREBP complex from the ER to Golgi by phosphorylating SREBP-1c and thus modulating its affinity for Sar1p and Sec23/24 at the expense of interactions with Insig (Yellaturu et al., 2009a,b). The latter may show increased degradation in the presence of insulin. Furthermore, a major downstream regulator of the PI3K/Akt pathway is mTORC1, which positively regulates SREBP processing (Porstmann et al., 2008), possibly by phosphorylating the S6 kinase (Owen et al., 2012). There is also an intriguing connection between SREBP-2 and the “activating transcription factor-6” (ATF6), an ER membrane-bound transcription factor that like SREBP is proteolytically cleaved at the Golgi. This proteolysis releases a transcription factor that binds to nuclear SREBP-2, down-regulating its gene expression (Zeng et al., 2004). Importantly, ATF6 is stimulated by the accumulation of misfolded or unfolded proteins during ER stress, which may occur in response to glucose deprivation (Brewer, 2014). Thus, in the absence of glucose, lipid and cholesterol biosynthesis may be downregulated to conserve energy. Other examples of metabolic pathways regulating SREBP include polyunsaturated fatty acids, glutamine, the NAD^+^-dependent deacetylase SIRT1, fibroblast growth factor 21, and retinol binding protein (Daemen et al., 2013). How these impinge on trafficking of SREBP is an open question.

Although now a well-established pathway, there is much work to be done to understand the genes targeted by SREBPs. In myotubes, greater than a thousand genes may be upregulated or downregulated in response to SREBP activation (Rome et al., 2008). Of note is the number of membrane trafficking-associated genes that are affected including the nominally epithelial specific μ1b adaptin, the clathrin light chain, the COPII coat protein component Sec23B, the v-SNARE VAMP3, the exocyst subunit Sec6, Rab33A, and a large number of microtubule/myosin motors to name a few (Rome et al., 2008). Why these particular genes are targeted and the specific membrane trafficking events they control in response to SREBP regulation remains to be determined. Finally, there is an increasing realization that SREBP-2 may also regulate a diversity of other cellular functions including autophagy, phagocytosis, membrane repair, and cell cycle control (Daemen et al., 2013).

### Lipid droplets

Lipid droplets (LDs) are an accumulation of lipid esters (triacylglycerides, steryl esters, and diacylglycerol) bound by a phospholipid monolayer that also contains cholesterol and peripheral proteins (Ohsaki et al., 2014; Pol et al., 2014). Because excess lipid accumulation is observed in obesity, diabetes, and atherosclerosis, the functions of LDs are of enormous interest. Moreover, LDs are linked to autophagy, and apparently play an important, albeit, ill-defined role in regulating the turnover of cellular lipids. Furthermore, pathogens such as hepatitis virus type C and *Chlamydia trachomatis* are thought to use LDs for their growth. LDs are present in all cells, but particularly rich in white adipocytes where they form one large droplet of a size >100 μm. In other cell types LDs are much smaller (~0.5–5 μm in diameter), and more numerous. There is some compositional heterogeneity in LDs, with some containing predominantly triglycerides (TGs), while others are enriched in steryl esters (Ohsaki et al., 2014). Peripheral proteins associated with LDs include the perilipins (1–5 isoforms); however, proteomic studies indicate the presence of more than 200 proteins, many of which are of unknown function in LD biology (Hodges and Wu, 2010). Kinesin and dynein are associated with LDs, likely explaining the long-range, microtubule-dependent movements of some of these organelles (Welte, 2009). LDs are closely apposed to the ER, their likely site of biosynthesis, as well as mitochondria and peroxisomes (Ohsaki et al., 2014); however, the mechanisms by which these organelles interact are not well-understood and open to further investigation. One possibility, described below, is that LDs form MCSs with proteins in the ER membrane.

An important, but unanswered question is the mechanism(s) of LD biosynthesis. Interestingly, LDs contain diacylglycerolacyltransferase 2 (DGAT2), an integral membrane enzyme that catalyzes the final steps of TG synthesis (Harris et al., 2011; Wilfling et al., 2013). The current model is that LDs are formed in the ER membrane (Ohsaki et al., 2014). As the concentrations of TGs or steryl esters increases to a few mole %, they are thought to aggregate, and become sequestered between the leaflets of the ER membrane, i.e., the nascent LD lumen. They may then bud off the ER membrane or may be formed as a result of maturation of ER-associated vesicles. Subsequent to their formation, LDs are thought to grow by a number of mechanisms including LD-LD fusion (apparently a rare event under most laboratory conditions), or TG transfer between closely apposed LDs, a function that depends on Perilipin-1, “fat storage-inducing proteins” FIT1/2, Fsp27, and local TG synthesis driven by DGAT2 (Chang et al., 2006; Gong et al., 2011; Sun et al., 2013; Wilfling et al., 2013).

The machinery that promotes early LD formation is the subject of active research. Using a genome-wide siRNA screen in Drosophila S2 cells, some 227 genes were identified that alter the morphology of LD formation (Guo et al., 2008). The 132 genes with marked phenotypes sort into five categories. Class I genes show reduced numbers of LDs and included the diacylglycerolacyltransferase protein Midway, and subunits of the spliceasome and proteasome. Class II genes result in smaller, dispersed LDs and included dynein and RNA polymerase II subunits. Class III genes show dispersed droplets that were a bit larger than normal. Intriguingly, the genes include members of the Arf1-COP1 vesicular transport machinery—a striking finding. Class IV genes result in clusters of LDs and Class V genes contained one or more very large droplets. Genes in the latter category include SREBP and SCAP.

Arf1-COP1 complexes are involved in retrograde transport from the Golgi apparatus to the ER, and also play roles at the TGN and in endosomes. COPII has also been implicated in LD biogenesis (Soni et al., 2009), but this finding is controversial (Guo et al., 2008). The function of Arf1-COPI in LD biogenesis is still being resolved. Initial studies indicated that Arf1-COP1 were involved in lipolysis, and consistent with this possibility is the observation that Arf1-COPI regulates the delivery of adipose triglyceride lipase to droplets (Soni et al., 2009; Ellong et al., 2011). Moreover, enzymes involved in LD biogenesis are also transported to LDs in an Arf1-COP1-dependent manner (Wilfling et al., 2014). How Arf1-COP1 promotes these transfers is a matter of conjecture. One possible model is that Arf1/COP1 acts to recognize cargoes and promote their targeting to LDs in a manner similar to how this coat functions in the biosynthetic pathway. However, a more recent analysis indicates that Arf1-COP1 directly associates with LDs, and may function to stimulate the budding of nano-droplets from their surfaces (Wilfling et al., 2014). In turn, these budding events are proposed to affect LD surface tension in a manner that promotes formation of MCSs between the LD and the ER. Once formed, the MCS then allows for proteins to transfer from the ER to the LD particle. It is worth noting that in these studies association of Arf1-COPI on LDs is highly discrete and focused (Wilfling et al., 2014), indicating that if Arf1-COP1 regulates nano-droplet formation, then it does so at localized sites on the LD. In addition to its effects on LDs, Arf1-COP1 could also regulate the ER lipid composition, and in doing so regulate the transfer of proteins/lipids at ER-LD MCSs.

Because of their association with metabolic disorders such as diabetes, studies of LD biogenesis, catabolism, and function are rapidly multiplying. Yet, there remains a large number of unresolved questions including how LDs are formed and the mechanisms of their turnover. For example, E2 and E3 ubiquitin ligases are associated with LDs (Ohsaki et al., 2014). Could their function be to regulate the degradation of LD-associated proteins? Somewhat unexpectedly, LDs also appear to play a role in protein degradation and sequestration (Ohsaki et al., 2014), as well as regulation of histone H2Av availability during development (Li et al., 2014). As noted above, Class I genes include subunits of the proteasome (Guo et al., 2008). Moreover, proteins such as ApoB, which is associated with nascent very low-density lipoprotein particles, accumulate in LDs when the proteasome is inhibited (Fujimoto and Ohsaki, 2006; Ohsaki et al., 2006), and the HMG-CoA reductase localizes to the LD fraction prior to extraction and degradation by the proteasome (Hartman et al., 2010). This indicates that for some proteins, their ER-associated degradation likely involves passage through LDs. However, the exact function of LDs in this regard is unknown.

### Pressing questions in the regulation of membrane traffic by cellular metabolism

Perhaps one of the grandest challenges in metabolism research today is to understand how nutrient status, lipid metabolism, membrane biogenesis, and organelle homeostasis are coordinated. Data thus far point to mTORC1/2 as the potential brains behind these operations as they receive sensory input from a number of external/internal sources and then transduce these signals by targeting signaling pathways that up- or down-regulate relevant membrane trafficking pathways. Thus, under conditions where metabolites are readily available, mTORC1/2 complexes act to decrease TFEB function (Palmieri et al., 2011; Roczniak-Ferguson et al., 2012), ensuring the cell does not devote excessive energy for degrading macromolecules in lysosomes or performing autophagy. Instead, more energy can be spent storing triglycerides in LDs, and by increasing the synthesis of sterols and lipids by way of SREBPs (Porstmann et al., 2008; Owen et al., 2012). The latter would allow the cell to match the increase in metabolic need with a corresponding increase in membrane biogenesis and traffic. In contrast, under nutrient poor conditions, mTORC1/2 activity is suppressed, leading to a stimulation of lysosome and autophagasome biogenesis, and an increase in triacylglycerol lipolysis (Soliman et al., 2010). Starvation is also likely to result in a general downregulation of membrane traffic.

If our premise is correct, then one of the big questions that needs to be addressed is the identity of the mTORC1 effector molecules and their targets. In the case of lysosomes and autophagasomes, TFEB is one effector. But, are there other effectors, including those that act more acutely? In the case of SREBPs, the target of the S6 kinase is not yet established, and it is unknown whether there are effectors other than the S6 kinase. Moreover, in the case of LDs and other membrane trafficking pathways there is a paucity of information about the identity of the mTORC1/2 effectors and targets. Furthermore, we have limited knowledge about the extent of the crosstalk in between the trafficking pathways, or how this cross-communication might be regulated. This is likely to be very important, because as noted above an increase in membrane traffic and organelle biogenesis must be coupled to increased availability of components such as lipids. Finally, an important goal is to understand how metabolic pathways are altered by disease, and how this impacts membrane traffic and the attendant changes in cell function that arise.

## How do organelles exchange content at membrane contact sites?

While membrane traffic often involves the movement of membranes and their cargoes via tubulovesicular carriers, MCSs are regions of close contact between organelles (less than 30 nm) that promote the exchange of Ca^2+^ and/or lipids (Helle et al., 2013; Prinz, 2014). Understanding how MCSs are formed, how they function, and how they are regulated is an additional challenge for our field. Formation of the MCS is thought to depend on tethering complexes, which bring the membranes in close proximity. In turn, these tethering complexes may recruit effector molecules that promote content exchange between organelles. However, in some cases tethering factors may also have effector function (see below). Reflecting its role as a major cellular site of lipid synthesis and Ca^2+^ storage, the ER forms MCSs with multiple organelles including: mitochondria, lysosomes, LDs, the Golgi apparatus, endosomes, and the plasma membrane (Helle et al., 2013; Prinz, 2014). However, we are likely to find other examples of inter-organellar interaction/communication as cell biologists look more closely. It is not known whether molecules other than Ca^2+^ and lipids are transferred at MCSs, but it is formally possible. Indeed, MCS-associated proteins are reported to have other functions including regulation of microautophagy, regulation of mitochondrial dynamics, and regulation of vesicular trafficking pathways (Helle et al., 2013). An example of the latter is described below.

### A plasma membrane-ER MCS that promotes Ca^2+^ uptake in response to store depletion

A variety of cellular processes including sensory perception, cell migration, muscle contraction, transcription, T-cell activation, and regulated secretion depend on the sustained release of Ca^2+^ from the ER, the major intracellular site of Ca^2+^ storage (Soboloff et al., 2012; Hooper et al., 2013). However, once the Ca^2+^ stores are depleted their replenishment depends on a process called “store-operated Ca^2+^ entry.” This function depends on two families of proteins: the stromal interaction molecules (STIM) 1/2, which act as Ca^2+^ sensors, and Orai1-3 (Soboloff et al., 2012; Hooper et al., 2013). The latter are plasma membrane-associated, Ca^2+^ release-activated channels (a.k.a. CRACs) that serve as conduits for Ca^2+^ entry into the ER. Mutations in STIM1 or Orai1 lead to severe immunodeficiencies, confirming the importance of these proteins in normal cellular function (Mccarl et al., 2009; Picard et al., 2009; Feske et al., 2010).

**Figure 3:**
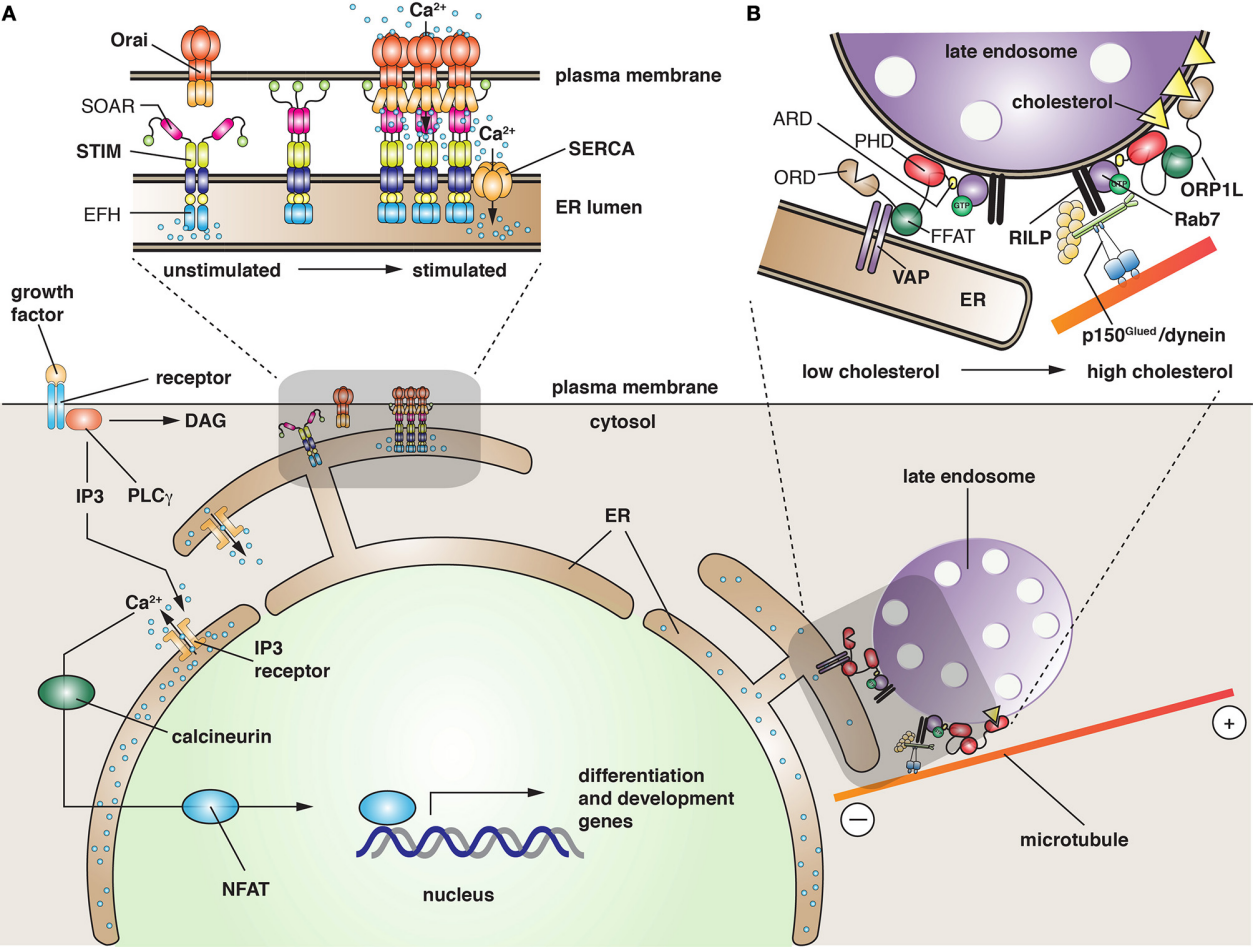
**Function of membrane contact sites in the cell. (A)** Ligand-bound growth factor receptor stimulates activation of phospholipase C-γ (PLC γ), which hydrolyzes phosphatidylinositol 4,5-bisphosphate into diacylglycerol (DAG), and inositol 1,4,5-trisphosphate (IP3). The latter binds to the ER-localized IP3 receptor, triggering the release of Ca^2+^ from ER stores. The released Ca^2+^ has several functions including activation of “nuclear factor of activated T cells” (NFAT), a family of five proteins that regulate transcription of genes required for development and differentiation. In response to a large stimulus or when long-term elevation of Ca^2+^ occurs, the ER stores become depleted. The change is sensed by the N-terminal, EF-hand domain (EFH) of STIM, resulting in the translocation of STIM to MCSs closely apposed to the plasma membrane. This relocalization is facilitated by a C-terminal polybasic tract of amino acids (green spheres in STIM structure) that promote interactions with lipids in the plasma membrane. Recruitment of Orai channels is potentiated by the “STIM-Orai activating region” (SOAR), a domain in STIM that is thought to induce a conformational change in Orai, leading to Ca^2+^ entry into the cell cytoplasm. The refilling of ER Ca^2+^ stores is likely facilitated by close apposition of SERCA with the STIM/Orai complex. **(B)** In the absence of cholesterol, late endosomes become less motile, collect at the cell periphery, and the LPT ORP1L promotes the formation of an MCS between the ER and the late endosome. ORP1L does so by binding the ER protein VAP (via its diphenylalanine in an acidic tract domain, FFAT), and simultaneously binding lipids (via its pleckstrin homology domain, PHD) and Rab7 (via its ankyrin repeat domain, ARD), a GTPase present on late endosomes. As cholesterol levels rise, possibly as a result of cholesterol transfer from the ER, the “oxysterol recognition domain” (ORD) of ORP1L binds cholesterol. This interaction disengages the link between the FFAT domain and VAP, and promotes the recruitment of the p150^Glued^ component of cytoplasmic dynein by way of the Rab7 effector RILP. When fully assembled, the dynein motor promotes minus-end directed movements of late endosomes, causing them to accumulate centripitally.

STIM1 is a type 1 transmembrane protein that is localized primarily at the ER. Its structure has been partially solved (Soboloff et al., 2012; Yang et al., 2012; Stathopulos and Ikura, 2013). At Its N-terminus lies a canonical Ca^2+^-binding EF-hand domain, which is located in the ER lumen, and works in conjunction with a non-canonical EF-hand domain and SAM (sterile α motif) domain to sense Ca^2+^ depletion. At resting luminal ER Ca^2+^ concentrations (~400 μM), STIM1 is distributed throughout the ER; however, as ER Ca^2+^ stores are depleted, STIM1 (which binds Ca^2+^ with a K_D_ of 200 μM) is presumed to undergo a large conformational change that leads to rapid oligomerization of the molecule (Figure 3A). This is coupled to translocation of STIM1 to an ER-plasma membrane MCS. This process depends, in part, on a poly-lysine tract at the C-terminus of STIM1 that allows for it to interact with lipids (Liou et al., 2007; Korzeniowski et al., 2009). The movement of STIM1 is followed by migration of Orai1-3 to the MCS, where it interacts with the STIM-Orai activating region (SOAR) found in the cytoplasmic domain of STIM1 (Yang et al., 2012) (Figure 3A). The Orai channels have four transmembrane domains with cytosolic N- and C-termini. The functional regions of the channel are being defined using both structural and mutational analyses (Yeromin et al., 2006; Mcnally et al., 2009; Feske et al., 2010; Lis et al., 2010; Zhang et al., 2011; Liu et al., 2012; Mcnally and Prakriya, 2012; Stathopulos and Ikura, 2013). The precise mechanisms by which Orai channels interact with STIM1 and how these interactions promote Orai activity will require further research. However, the interaction is likely to be direct as recombinant STIM cytosolic fragments are sufficient to increase Ca^2+^ import in yeast expressing Orai channels (Zhou et al., 2010). Other work indicates that the C-terminal tail of Orai1 contains a cluster of acidic residues that may interact with basic amino acids in the SOAR domain of STIM1 (Calloway et al., 2009).

One mystery is how Ca^2+^ entering the Orai channel is funneled into the ER and prevented from rapidly diffusing into the cytosol. A possible mechanism is the localization of the sarcoplasmic reticulum/ER Ca^2+^ ATPase (SERCA) to the ER-plasma membrane MCS (Figure 3A). SERCA is responsible for transporting cytosolic Ca^2+^ into the ER against a steep concentration gradient. Interestingly, SERCA appears to associate with STIM1/Orai1 (Lopez et al., 2008; Manjarres et al., 2010), indicating that MCS-localized SERCA may be critical for refilling ER stores. Furthermore, it demonstrates that STIMs and perhaps Orai1 channels may recruit effectors that can regulate the process of Ca^2+^ exchange between these two membranes. This appears to be true of other MCSs as well, and various effectors have been proposed (Helle et al., 2013).

How the ER and plasma membrane are tethered at the STIM1/Orai1 MCS is not known. One possibility is that STIM and Orai act as a tethering complex; indeed, overexpression of STIM1 leads to increased numbers of ER/PM contact sites (Wu et al., 2006; Lur et al., 2009). Moreover, TIRF studies indicate that STIM1 may cycle at defined ER-PM MCSs (Smyth et al., 2008). But if true, then it becomes difficult to envision how ER-PM MCSs are maintained and reused, unless a small number of STIM and Orai molecules remain at contact sites and form a nidus for expansion of the MCS upon Ca^2+^ depletion. In addition to STIM1, tagged-Orai1 also appears to traffic to ER-PM MCSs (Woodard et al., 2008); however, if its Ca^2+^-dependent redistribution reflects vectorial traffic or diffusion in the plane of the plasma membrane remains to be determined. Moreover, endogenous Orai1 may not undergo the same degree of clustering as observed for overexpressed, tagged versions of Orai1 (Hong et al., 2011), indicating the degree of clustering at MCSs may be overestimated using overexpression systems.

### Non-vesicular lipid exchange at MCSs

In addition to Ca^2+^ exchange, MCSs are also sites of non-vesicular lipid exchange (Helle et al., 2013). This exchange is mediated by lipid transport proteins (LTPs), which bind a variety of lipid species via their lipid transport domains. Indeed, many LTPs can bind two lipid species in a mutually exclusive manner. For example, the ceramide transporter CERT binds either ceramide or diacylglycerol, and thus may promote lipid exchange (Kudo et al., 2008). LTPs are targeted to MCSs by way of specific interaction domains that allow these molecules to bridge organellar compartments. For example, CERT and some “oxysterol-binding protein-related proteins” (ORPs) such as ORP1L have an FFAT (diphenylalanine in an acidic tract) motif, which interacts with the ER resident protein VAP (VAMP-associated protein) (Loewen et al., 2003) (Figure 3B). Moreover, these proteins have a pleckstrin homology domain (PHD), which binds to specific phosphoinositides on the Golgi and plasma membrane (Lemmon and Ferguson, 2001).

LTPs are important components of MCSs that form between the yeast vacuole and nuclear ER (Osh1/Scs2-22 and Vac8/Nvj1), the Golgi and ER (CERT and VAP), and the LD. In the latter case, the LD enzyme DGAT2 is thought to interact with FATP1, an acyl-CoA synthase that is present in the ER membrane (Xu et al., 2012). An additional example is the MCS that forms between Neiman Pick C protein-positive late endosomes and the ER (Van Der Kant et al., 2013). The MCS in this case is likely formed by VAP and ORP1L, and the cholesterol transfer that ensues may be important for the biogenesis of the intraluminal vesicles that form in the lumen of late endosomes/multivesicular bodies (Rocha et al., 2009). MCS formation between the ER and late endosomes is only observed when cholesterol levels in the cell are low, a change that is apparently detected by ORP1L's ORP-related domain (ORD), a conserved domain found in ORP family members (Helle et al., 2013). Because late endosomes move centripetally, where they accumulate in a peri-centriolar distribution, there must be a mechanism to accommodate these movements during MCS formation. Interestingly, ORP1L has an additional function in this system, one that uncouples late endosomes from microtubules (Figure 3B). Under conditions where cholesterol is replete, Rab7 recruits the “Rab7-interacting lysosomal protein” (RILP), which associates with the p150^Glued^ subunit of the microtubule dynein motor complex (Rocha et al., 2009). As cholesterol levels drop, binding of ORP1L's FFAT domain to VAP results in dissociation of dynein and a redistribution of the late endosomes toward the periphery. One possibility is that the decrease in centripetal motion facilitates the transfer of cholesterol at the MCSs.

### Open questions in MCS biology

There are a significant number of unknowns in the MCS field, ensuring that analysis of these interactions will be an active area of research for years to come. For example, Helle et al. have defined a number of reasonable criteria that would establish a *bona fide* tether, but these criteria have not been met for the majority of putative tethering complexes (Helle et al., 2013). Moreover, LTPs are though to interact with two organelles simultaneously, but it is not clear whether this serves as a mechanism to specify their localization at the MCS or whether it serves to tether membranes at these sites of interaction. Furthermore, there are reactions in the cell that are likely to depend on MCSs, but the critical components have not been identified. For example, it has been known for decades that *de novo* synthesis of phosphatidylethanolamine (PE) and phosphatidylcholine (PC) requires a close interaction between the ER and mitochondria. While phosphatidylserine (PS) is synthesized in the ER by the PS synthase, the formation of PE is dependent on a decarboxylase that resides in the inner mitochondrial membrane. Finally, PE is converted in the ER by a series of methylation steps to PC, which requires the activities of the phosphocholine cytidylyltransferase enzymes Cct1 and Cct2. Despite the importance of these reactions, the LTPs and tethering factors remain unknown (Helle et al., 2013). Interestingly, Cct1 and Cct2 associate with LDs when triglyceride precursors are fed to cells, indicating that LDs may play a critical role in this process (Krahmer et al., 2011). Furthermore, it indicates the PC synthesis may require the formation of multiple MCSs, but how these are regulated and coordinated is unknown. Additional questions in MCS biology and function include: Are MCSs dynamic or stable, and how does this affect their functions? Since organelles such as the ER form numerous contacts, how does the activity of one MCS affect the activity of others? How important is non-vesicular vs. vesicular lipid transport in the cell?

## How is membrane traffic exploited during complex processes such as development?

An additional challenge for our field is to understand how the complex events that occur during development are intimately linked to exocytosis and endocytosis. Development is a process whereby a single cell undergoes cell division, its progeny differentiate into a number of specialized cell types, and these differentiated cells form higher order structures (e.g., organs) that allow for specialized functions. We consider two types of mechanisms where membrane traffic is critical to achieving cell differentiation: one involves cell surface receptor-ligand pairs and the other depends on the formation of a concentration gradient. However, other developmental processes can be considered as well, including cell migration, apoptosis, autophagy, lumen formation for organ ducts, and processes such as epithelialization and morphogenesis. Indeed, most, if not all, developmental processes are likely dependent on membrane traffic.

### Notch signaling and the generation of cellular asymmetry

Notch signaling is a pathway for cell-cell communication that occurs during development and promotes the formation of tissue boundaries, differentiation of cells arising from equipotent precursors, and generation of different cell types after a single asymmetric cell division (Furthauer and Gonzalez-Gaitan, 2009; Yamamoto et al., 2010). It is not limited to embryonic development, as it also occurs during any process that involves differentiation of precursor cells including normal tissue turnover as well as regeneration in response to injury. The following discussion is adapted from a recently published monograph (Apodaca et al., 2012).

**Figure 4:**
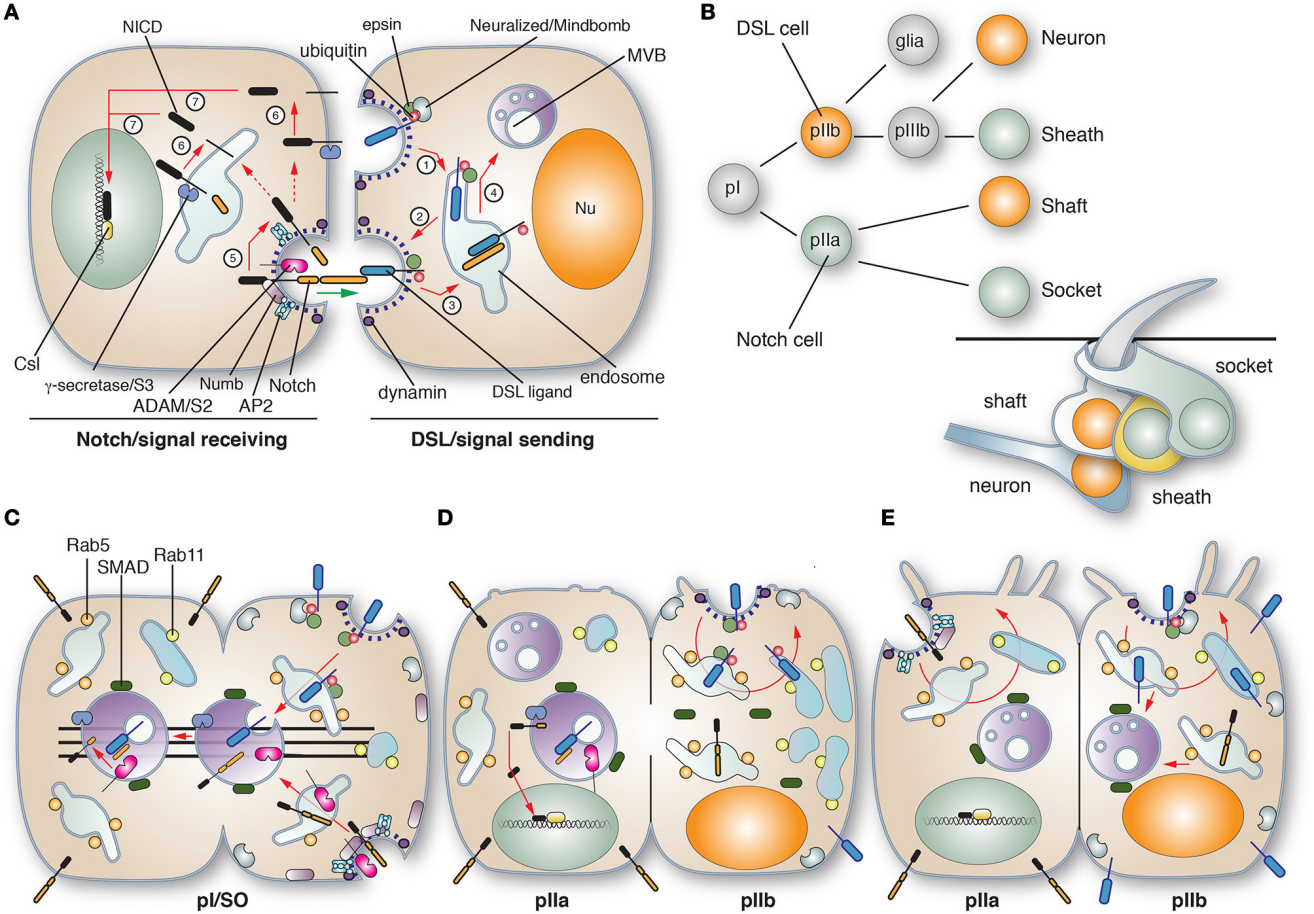
**Role of membrane traffic in Delta-Notch signaling. (A)** In canonical Notch signaling the signal-sending cell expressing Delta/Serrate/Lag2 (DSL) ligands interacts with the Notch receptor on the signal-receiving cell, ultimately leading to Notch-dependent changes in gene expression. On the signal-sending cell, DSL ligands are ubiquinated by the action of the E3 ligases Neuralized or Mindbomb. The ligand is endocytosed in an epsin and dynamin-dependent manner (step 1), and then delivered to early and recycling endosomes, from which the ligand can recycle to the cell surface (step 2). The DSL ligand encounters Notch on the signal receiving cell and the endocytosis of the ligand results in a pulling force (marked with a green arrow) that dislodges the previously cleaved N-terminal extracellular domain of Notch (the product of S1 cleavage) into the signal-sending cell, promoting its “trans-endocytosis” (step 3), delivery to MVBs (step 4), and then likely degradation in lysosomes (not shown). The pulling force generated during ligand endocytosis triggers cleavage of Notch on the signal-receiving cell by an ADAM metalloproteinase (S2 cleavage; step 5). This is followed by S3 cleavage, which is mediated by the transmembrane γ-secretase (step 6). The latter releases the Notch intracellular domain (NICD), which enters the nucleus and associates with CBF1/Su(H)/Lag1 CSL family transcriptional cofactors to activate target genes. The sites of S2 and S3 cleavage are unclear, but may occur at the cell surface or within endosomes. Notch signaling depends on dynamin and Numb, an adaptor that links Notch to the α-adaptin of the AP2 adaptor complex. **(B)** Cell lineage of the Drosophila mechanosensory bristle. pI cells, also called single organ precrusors (SOPs), undergo asymmetric cell division to give rise to pIIa and pIIb cells, which express DSL ligands or Notch, respectively. The pIIb cell gives rise to a glial cell (which undergoes apoptosis) and a pIIIb cell that like pI will give rise to a DSL ligand-expressing neuron and Notch-expressing sheath cell. The pIIa cell gives rise to the DSL ligand-expressing shaft cell and the Notch-expressing socket cell. The organization of the mechanosensory bristle complex is shown in the bottom right. **(C–E)** Membrane trafficking events leading to the asymmetric cell division of the pI cell. **(C)** During division of the pI cell, Neuralized and Numb are segregated into the emerging pIIb cell by the action of the Par complex (not shown). Neuralized catalyzes ubiquitination of the DSL ligand, promoting its dynamin- and epsin-dependent endocytosis and delivery to Rab5-positive early endosomes and then SARA-positive MBVs. Likewise, Notch is internalized in an AP2-, dynamin-, and Numb-dependent manner, and then trafficked via Rab5-positive endosomes to SARA-positive MVBs. Prior to cytokinesis, the SARA-positive endosomes containing DSL ligand and Notch align along the central spindle (shown as lines in the figure) and partition into the emerging pIIa cell. During the transition to cytokinesis the DSL ligand and Notch assume an anti-parallel orientation in the forming SARA-positive MVB, stimulating S2 cleavage. See Furthauer and Gonzalez-Gaitan (2009) for an alternative mechanism of Notch activation in MVBs. **(D)** As cytokinesis proceeds S3 cleavage occurs, releasing the NICD. By this point the majority of SARA-positive MVBs have segregated into the pIIa cells, while Rab11-positive recycling endosomes are seen to organize at the centrosomal region of the pIIb cell. The concentration of these endosomes likely ensures that internalized DSL ligands are recycled and not targeted for degradation. **(E)** Asymmetric cell division is complete. The endocytic organelles take up a somewhat random distribution in the cytoplasm and in the pIIb cells SARA-positive MVBs reform using the cytoplasmic pool of SARA that partitions equally during cytokineses. Notch present in the pIIb cells is likely targeted to MVBs for degradation. Figure and legend used with permission and is from Apodaca et al. (2012).

Somewhat akin to SREBP signaling, Notch signaling requires intramembranous proteolysis and release of a peptide fragment that acts as a transcription factor. The process is initiated when a membrane-bound Delta/Serrate/Lag2 (DSL) ligand on the surface of a signal-sending cell binds to the membrane bound Notch receptor on a signal-receiving cell (Figure 4A). This triggers the proteolysis of the extracellular, juxtamembrane region of Notch by a membrane-bound ADAM family proteinase. This is rapidly followed by further cleavage of the intramembrane domain by the γ-secretase, promoting the release of the Notch intracellular domain (NICD). This peptide fragment is transported into the nucleus and triggers transcription upon its association with CBF1-Su-Lag1 (CSL) family cofactors. Changes in gene expression in the Notch-expressing, signal-receiving cell leads to cellular asymmetry.

In addition to exocytosis, which ensures the surface delivery of Notch, Delta, ADAM, and γ-secretase, Notch signaling is also dependent on endocytosis (Yamamoto et al., 2010). In the case of Notch, and its co-factor Sanpodo, internalization requires Numb, an adaptor that links the receptor to the AP2 adaptor complex. The function of Notch endocytosis is not completely clear, but may serve to “activate” the receptor prior to proteolysis, or it may be required to promote Notch proteolysis and signaling (see text below). Delta is also endocytosed, but in a process that depends on the E3 ubiquitin ligase Neuralized or Mindbomb, as well as the adaptor protein epsin (“liquid facets” in fly speak), which binds to ubiquitinated cargoes (Wang and Struhl, 2005). One model suggests that internalization is required to “activate” Delta, but the nature of this activation is not well-understood and recent studies indicate that endocytosis and recycling of the Delta ligand do not affect its affinity for, or interactions with Notch (Shergill et al., 2012). In epithelial cells, Delta may need to be transcytosed and thus changes in ligand distribution or its association with specific membrane domains may be critical for its function. Why ubiquitin promotes recycling/transcytosis and not degradation, and the signals in Delta ligands that specify apical targeting are open questions. An additional endocytosis-requiring step is one that occurs upon Delta binding to Notch. In this case, “pulling” of the ligand by the endocytic machinery may cause a conformational change in Notch, triggering its proteolysis, and uptake of the Notch extracellular domain by Delta cell in a process termed “trans-endocytosis” (Figure 4A). In a recent study, optical tweezers were used to show that upon receptor binding, Delta ligands indeed exert a pulling force, and this force generation is dependent on the activity of Mindbomb, dynamin, epsin, and actin (Meloty-Kapella et al., 2012). The fate of the Notch extracellular domain and the effect of it on Delta traffic are not well-understood.

Intriguingly, membrane traffic also plays a critical role in asymmetric cell division, which generates different cell types after a single mitotic event. This process requires careful coordination of membrane traffic, cell polarization, cytokinesis, organellar movements, and changes in gene expression. Asymmetric cell division occurs during the formation of the four cell types that give rise to the mechanosensory bristles that cover the dorsal thorax of the fly pupae (Meloty-Kapella et al., 2012). These include the neuron, sheath, shaft, and socket cells (Figure 4B). In the sensory organ precursor cells (SOPs, also known as pI), which express both Delta and Notch, mitosis is accompanied by endocytosis of Delta and Notch (Figure 4C). In both cases the required endocytosis machinery is partitioned into the emerging pIIb cell: Neuralized along with epsin in the case of Delta (Emery et al., 2005), and Numb and AP2 in the case of Notch (Tong et al., 2010).

Apparently, Delta is initially delivered to SARA (Smad Anchor for Receptor Activation)-positive MVBs (Figure 4C), but its fate changes as cell division proceeds and Rab11a-positive endosomes are established in the centrosomal region of the nascent pIIb cell (Figure 4D). The Rab11-positive endosomes are thought to stimulate Delta recycling, preventing its delivery to MVBs and rapid degradation (Emery et al., 2005). In contrast, Notch and its cofactor Sanpodo are primarily targeted to the forming SARA-positive MVBs, which segregate upon cell division into the pIIa cells (Coumailleau et al., 2009) (Figure 4D). However, after cytokinesis, these endosomes re-form in pIIb cells from the cytoplasmic pool of SMAD (Figure 4E). In pIIb cells both Notch extracellular and intracellular domains are found in SARA endosomes. However, in the SARA-positive endosomes of nascent pIIa cell, the extracellular domain of Notch, but not its intracellular domain, are present, indicating the Notch activation has occurred concurrent with cell division (Figure 4D) (Coumailleau et al., 2009). The question is where did the Notch activation occur? If at the cell surface, it may indicate that the NICD was released *en route* to or within the SARA endosomes. However, an alternative possibility is that Delta and Notch differentially partition into the limiting membrane of the SARA MVB or the intraluminal membranes. This would then allow for a productive antiparallel interaction that would promote Notch cleavage and release of its intracellular domain (see Figure 4D). Consistent with this model are previous studies that show Notch signaling is dependent on ESCRT complex proteins (Vaccari and Bilder, 2005). Regardless of the site of activation, these studies emphasize the critical role that vectorial membrane traffic in promoting Notch signaling and cell differentiation. Furthermore, the discussion above indicates that even in this well-studied system, a number of unanswered questions remain.

### Morphogen gradients

An additional developmental mechanism that affects cell differentiation is the formation of morphogen gradients (Erickson, 2011; Rogers and Schier, 2011; Bokel and Brand, 2013). In these gradients, a cellular population secretes a mediator (i.e., morphogen), which moves outwards and affects the developmental fate of adjacent cells as a function of their position in the gradient. Because not all gradients are identical in their range or effects, important questions include: how the gradient is formed, how it is shaped, and how cells are able to differentially respond to the concentration of morphogen? Early models posited that the shape of the gradient is determined primarily by the rate of morphogen secretion and its diffusion (Turing, 1990). However, other investigators argued that diffusion alone cannot explain how a gradient of cell fates is established, as diffusion ultimately leads to the saturation of all cells with morphogen (Wolpert, 1969). Furthermore, simple diffusion models are not thought to explain why some morphogens can undergo rapid diffusion in all directions, but the gradients they generate form slowly, directionally, and involve both extracellular and intracellular gradients (Rogers and Schier, 2011).

To explain these later observations, investigators have proposed a restricted diffusion model, which posits that morphogen spreading is affected by interactions with cognate receptors, or interactions with extracellular matrix components. For example, the secreted morphogen fibroblast growth factor (FGF, which has 22 isoforms in mammals) interacts with heparin sulfate (and other sulfated oligosaccharides), which alters the rate and extent of diffusion (Bokel and Brand, 2013). Furthermore, some morphogens such as BMP interact with potentiators and/or inhibitors, and in some cases morphogens are lipid modified or found within the cell cytoplasm of syncitia (Erickson, 2011).

In addition to the above models, it is now established that endocytosis is key to gradient formation and cellular responses to morphogens. In the prevailing synthesis-diffusion-clearance (SDC) model, morphogens are secreted and then diffuse outwards. The shape of the gradient is then determined by the clearance, i.e., degradation, of the morphogen and its cognate receptor (Wartlick et al., 2009). In this model, the higher the rate of diffusion and the lower the clearance rates, the shallower the gradient will be. Studies of FGF gradients provide evidence in support of such a model. In this case, blocking endocytosis by inhibiting dynamin results in a less steep gradient, extends the FGF lifetime (i.e., blocks its degradation), and increases the cellular zone where target gene expression is upregulated (Yu et al., 2009a; Nowak et al., 2011). In contrast, increasing endocytosis by expressing Rab5c causes a steeper gradient and reduces ligand half-life. While lysosomal degradation is an integral part of these models, there are few experiments that directly examine how lysosomal targeting and degradation (well-understood and highly regulated processes) impact morphogen gradient formation.

Other models for gradient formation, include the “active transport model,” in which morphogens are endocytosed and then “re-secreted” by a so-called “planar transcytosis mechanism” (Entchev et al., 2000; Entchev and Gonzalez-Gaitan, 2002). The exocytosed morphogen is taken up by an adjacent cell, which can repeat the process. Such a mechanism would allow gradients to form relatively slowly, and across cell layers, as well form morphogen gradients intracellularly and extracellularly. This model may explain some facets of the anterior/posterior gradient formed by the TGF-β homolog Decapentaplegic (Dpp) in the *Drosophila* wing disc, a flat epithelial pouch that will give rise to the wing and its associated structures. Consistent with an important role for endocytosis in gradient formation, the majority of Dpp in the signal receiving cells is found in endosomes (Entchev et al., 2000). Internalization of Dpp is reported to be dependent on the Dpp receptor Thickveins, and Dpp gradients are dependent on Rab5 function (Entchev et al., 2000). Intriguingly, and unlike similar experiments performed with FGF, no gradient forms across cells that express a temperature-sensitive mutation of the dynamin homolog *Shibire*. Moreover, Dpp-GFP fails to move across intervening clones of dynamin-defective cells, leaving a “shadow” in their wake where no gradient is formed even though these adjacent cells express Shibire (Entchev et al., 2000). At present there is limited genetic or morphological evidence showing that recycling or planar transcytosis is occurring or that reuptake by adjacent cells is required for gradient formation. Furthermore, the function of planar transcytosis in Dpp gradient formation has recently been questioned (Zhou et al., 2012b). The shape of the Dpp gradient is also affected by Rab7 overexpression, or by Rab7 mutants that stimulate degradation (Entchev et al., 2000). In this case, the gradient of activation was made shallower. Thus, regardless of the mechanism of morphogen transfer, a role for morphogen degradation is still likely to be key for shaping the gradient. If planar transcytosis also occurs, then it indicates that changes in the regulation of recycling vs. degradation may have critical roles in regulating the shape and reach of the gradient.

An important consequence of forming a morphogen gradient is that cells within the gradient are exposed to different concentrations of the morphogen. By responding to distinct concentration thresholds, the target cell then modulates the expression of different target genes. A well-known function of endocytosis in general, and endosomes in particular, is to regulate signal transduction (Gonnord et al., 2012). In the case of TGFβ, the mammalian ortholog of Dpp, the internalized receptor-TGFβ complex is delivered to endosomes where they encounter the FYVE-domain containing protein SARA (Murphy, 2007), which as described in the previous section is associated with multivesicular/late endosomes. This protein then recruits the transcription factor Smad2, which traffics to the nucleus upon release from the endosome. Thus, future studies are assuredly going to examine endosome dynamics and how they contribute signaling events, which not only affect gradient formation but differential gene expression and cell fate.

## How is membrane traffic used by highly differentiated cells to perform specialized cell functions?

Much of what we know about membrane traffic comes from work performed in workhorse cell lines like CHO, HeLa, and HEK, primary cell cultures of neurons, and genetically tractable cell types such as yeast. However, these cells do not reflect the diversity of cell types found in the body, nor the specialized cell functions that are performed by these highly differentiated cell types. Moreover, there is significant variation in the tissue-dependent expression of Rabs (including highly related Rab isoforms and housekeeping ones), effectors, and SNARE proteins—proteins required to form and regulate membrane trafficking pathways (Gurkan et al., 2005). Thus, it is likely that there are Rab-dependent trafficking pathways that have not been adequately studied, yet are critical for normal tissue function and body homeostasis. A challenge for the future is to understand the diversity of membrane trafficking events in specialized cell types, the regulation of these pathways, and how these trafficking pathways contribute to specialized cell function. Below, we provide just two examples of cell types where membrane traffic plays important functions that are only now being explored.

### Umbrella cells

**Figure 5:**
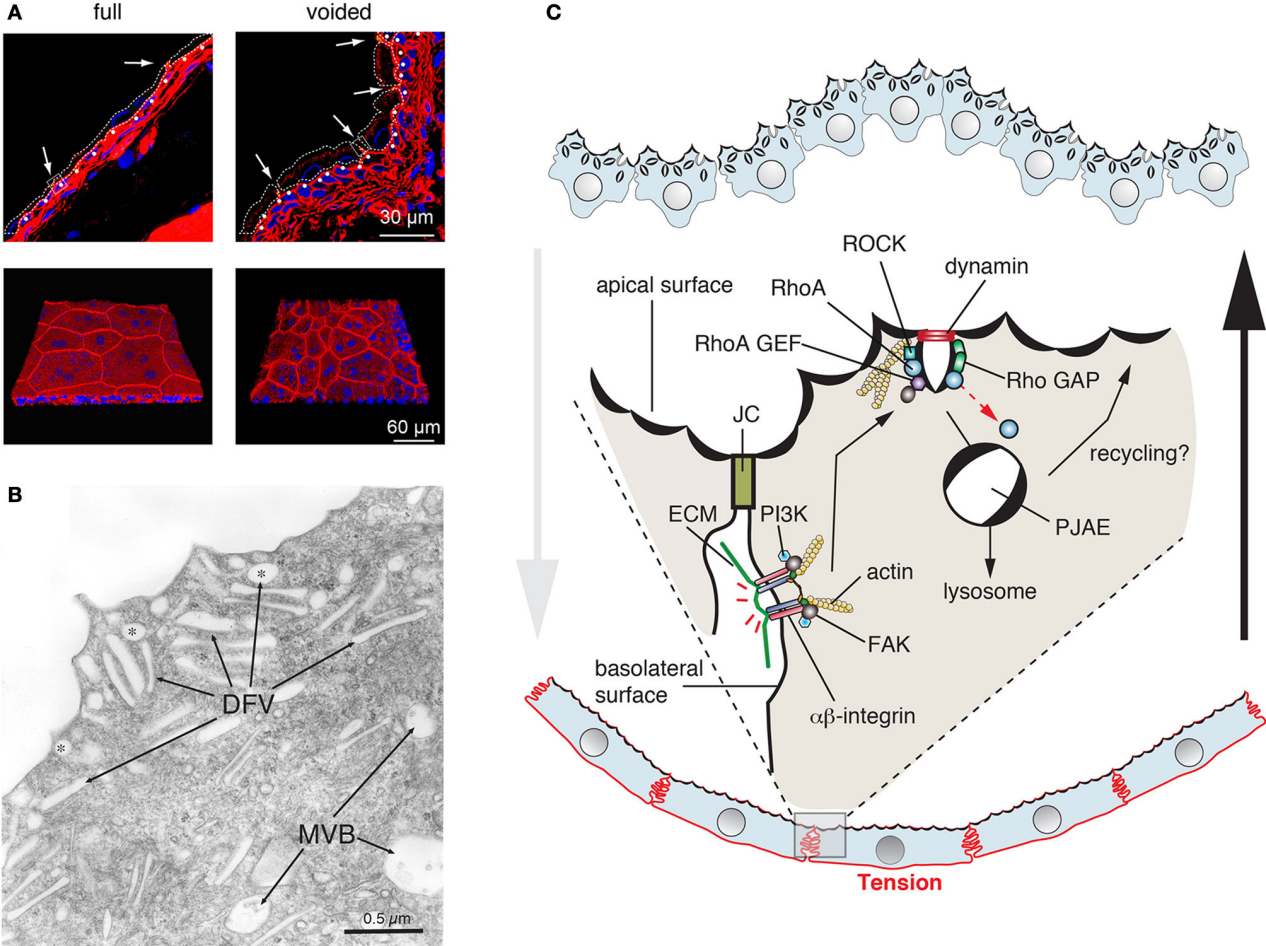
**Dynamic cell shape and apical surface area changes in bladder umbrella cells. (A)** Cell shape changes in the urothelium that accompany bladder filling and that occur after voiding. The upper panels are cross-sections of frozen bladder tissue and the bottom panels are a 3D reconstruction of whole-mounted tissue viewed *en face*. Cells are stained with rhodamine phalloidin (red), which labels the cortical actin cytoskeleton, and ToPro-3 (blue), which labels nuclei. In the upper panels, the approximate cell borders of the umbrella cells are indicated by dashed lines. The position of the junctional complex is marked with an arrow and the first layer of intermediate cells is indicated by closed, white circles. Images are used with permission and from Carattino et al. (2013) **(B)** Apical cytoplasm of umbrella cells showing discoidal (marked with asterisks) and fusiform shaped vesicles (DFV). Multivesicular, late endosomes (MVB) are also shown. Figure is used with permission and is from Khandelwal et al. (2009) **(C)** Model for regulation of clathrin-independent endocytosis in umbrella cells. In response to voiding, tension is increased across the serosal surfaces of the umbrella cell as the bladder smooth muscle contracts and actively refolds the mucosal surface of the bladder. This tension is sensed by basolaterally localized β1-integrins, which in response to tension stimulate the activation of the PI3K and focal adhesion kinase (FAK). In turn, these kinases stimulate the activity of RhoA, which likely acts through the Rho kinase (ROCK) to stimulate rearrangements of the actin cytoskeleton that promote dynamin-dependent endocytosis. The internalized apical membrane proteins are delivered to peripheral junction-associated endosomes (PJAEs), and then ultimately to late endosomes/lysosomes. Some recycling may occur from PJAEs.

Umbrella cells are polarized epithelial cells that form the outermost cell layer of the urothelium, the stratified tissue that lines the lower urinary tract including the kidney pelvis, ureters, and bladder (Khandelwal et al., 2009). Because the bladder forms the equivalent of a hollow bag, it is possible to open it and study the urothelium *ex vivo*, making it a particularly attractive model for cell studies. An important feature of the urothelium is that it must accommodate large changes in tissue stretch as urine is propelled from the renal pelvis to the ureters, and as the urine accumulates into the bladder prior to voiding. The umbrella cell has a number of features that allow it to adjust to this dynamic mechanical environment (Khandelwal et al., 2009). One is the ability of the umbrella cell to undergo large cell shape changes: it is somewhat cuboidal-shaped in the empty bladder, but highly flat and squamous in the filled bladder (Figure 5A). Second, the tight junction ring significantly increases in diameter, and returns to baseline within 5 min of voiding (Carattino et al., 2013). Third, and most relevant to this article are the dynamic changes in apical surface area. In response to bladder filling, the apical surface area dramatically increases (more than 100%), which results from the regulated exocytosis of an abundant population of subapical discoidal- and/or fusiform-shaped vesicles (DFVs) (Lewis and De Moura, 1982; Truschel et al., 2002; Khandelwal et al., 2008; Yu et al., 2009b) (Figure 5B). Upon voiding, the added apical membrane is then rapidly endocytosed, via a clathrin-independent pathway (Khandelwal et al., 2010).

Like other exocytic events, release of the DFVs is dependent on SNAREs, Rab GTPases including Rab11a, Rab8a, and their effector MyoVb (Born et al., 2003; Khandelwal et al., 2008, 2013). Rab27b and the “myelin and lymphocytic protein” (MAL) are also likely to play a role in these events (Chen et al., 2003; Zhou et al., 2012a). While Rab11a and Rab8a may act within a cascade (Khandelwal et al., 2013), the relationship between these Rabs and Rab27b is not clear. In response to bladder filling DFV exocytosis occurs in two stages (Balestreire and Apodaca, 2007; Yu et al., 2009b). Early stage exocytosis occurs first and requires fusion of a preexisting pool of DFVs. It is triggered by an apical non-selective cation channel, which is likely stretch sensitive and conducts Ca^2+^ into the cell (Yu et al., 2009b). The other, late-stage pathway is initiated when the bladder reaches its filling capacity and likely occurs in response to excess stretch. This stage requires protein synthesis and new secretion and is triggered in response to HB-EGF cleavage, EGF receptor transactivation, and ERK activation (Balestreire and Apodaca, 2007). It is unknown if the requirement for protein synthesis reflects new DFV formation or synthesis of proteins that are required for DFV exocytosis. An intriguing question, but one that still remains unanswered is how DFVs are formed. The classical model proposes that DFVs are a recycling pool of vesicles, but recent studies indicate that the major fate of apically internalized membrane is delivery to lysosomes (Khandelwal et al., 2008, 2010). Other studies indicate that DFVs are unlikely to be lysosome-related organelles (Guo et al., 2009).

A striking aspect of umbrella cell biology is the rapid recovery of cell shape and the decrease in apical surface area that accompanies voiding. Intriguingly, umbrella cells lack clathrin-coated pits or caveolae at their apical surfaces, indicating that apical endocytosis may occur exclusively by a clathrin- and caveolar-independent pathway. Such pathways have been described for several years but are not well-understood, because they can lack specific cargoes, carriers, inhibitors, or machineries (Mayor and Pagano, 2007; Sandvig et al., 2008; Hansen and Nichols, 2009), and some are only revealed when other forms of endocytosis are inhibited (Damke et al., 1994). The pathway in umbrella cells is related to the RhoA pathway described for the IL-2 receptor (Lamaze et al., 2001; Khandelwal et al., 2010). Both pathways require dynamin, RhoA, actin, and cholesterol, but not clathrin. Moreover, a related RhoA-dependent, but clathrin-independent pathway has been identified in yeast, but the cargoes and function of this pathway has not yet been described (Prosser et al., 2011). One intriguing aspect of the yeast pathway is that it is dependent on formins, proteins that regulate the actin and microtubule cytoskeleton (Prosser et al., 2011). But, it is not known if the RhoA pathway in multicellular organisms requires these proteins. The current dogma is that regardless of the pathway of entry, most cargoes internalized by clathrin-independent pathways are transported to Rab5- and EEA1-positive endosomes (Mayor et al., 2014). However, in umbrella cells apically internalized cargoes are delivered to EEA1-negative carriers, called peripheral junction-associated apical endosomes (PJAEs), that are ZO-1 positive and appear to deliver internalized apical membrane to late endosomes/lysosomes (Khandelwal et al., 2010). This indicates that differentiated cells may use pathways that are distinct from those found in fibroblasts and their ilk.

Of particular interest, is the finding that like exocytosis, endocytosis is also triggered by stretch, but apparently in response to refolding of the epithelium that occurs in response to contraction of the smooth muscle. The machinery that senses this refolding includes the β_1_-integrin, which is localized to the basolateral surface of the umbrella cells. In response to stretch, the β_1_-integrin appears to signal through phosphatidylinositol-3-kinase (PI3K) and the “focal adhesion kinase” (FAK) to stimulate RhoA activation (Khandelwal et al., 2010) (Figure 5C). Whether other molecules act upstream of the integrin, the mechanisms by which FAK/PI3K activate RhoA, and what RhoA does to potentiate rapid endocytosis are all unknown at this time. Interestingly, RhoA-dependent phagocytosis is also dependent on β_1_-integrin function (Dupuy and Caron, 2008), indicating some conservation between these two clathrin-independent pathways for endocytosis.

### T cells and the immunological synapse

**Figure 6:**
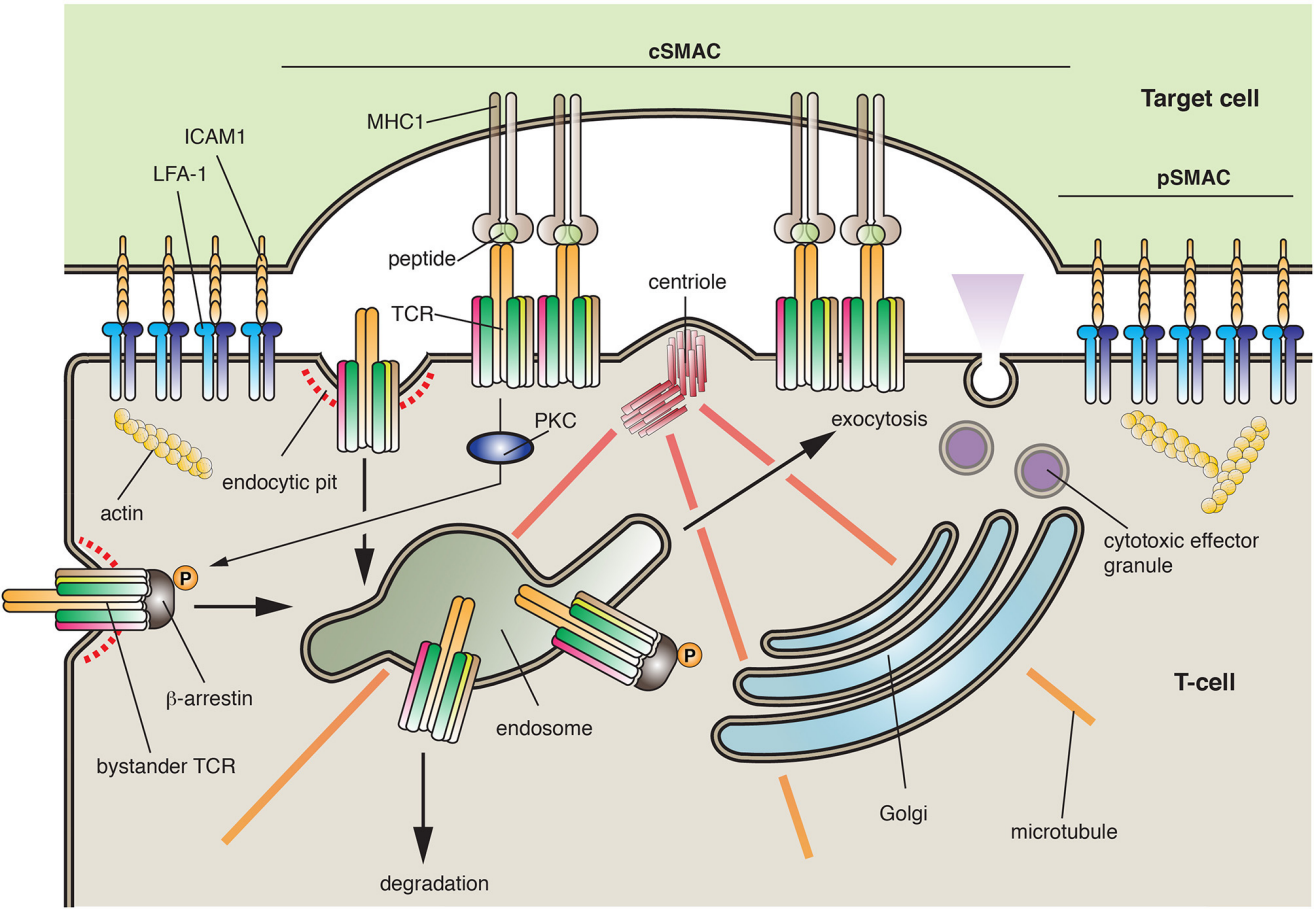
**The immunological synapse**. Immunological synapses are formed between T-cells and their cellular targets. The central supramolecular activation complex (cSMAC) is comprised of the T-cell receptor (TCR), its co-receptors, and its associated signaling proteins (most of which are not shown). In contrast, the peripheral SMAC (pSMAC) is formed by interactions between ICAM1 and LFA-1, which bring the cells in close proximity. During synapse formation, the centrioles are repositioned to just below the T-cell synaptic membrane, prompting the recruitment of endosomes and the Golgi to this region of the cell. The synapse allows for the recognition of antigen by the T-cell and in the case of cytotoxic T-cells forms a focused site for the release of cytotoxic effector granules. During synapse formation the TCR is internalized by clathrin-dependent endocytosis or by phagocytosis (not shown). The receptor can then be targeted to late endosomes/lysosomes for degradation. To ensure a constant supply of TCRs, activated T-cells promote the proteins kinase C (PKC)-dependent phosphorylation of β-arrestin, which interacts with “bystander TCRs” not present in the synapse. Bound to β-arrestin, the bystander TCR is endocytosed and then delivered by transcytosis to the synaptic membrane domain where it can extend the period of signaling within the synapse.

T-cells are a population of lymphocytes that are critical for cell-mediated immunity. They characteristically express the T-cell receptor (TCR), a multicomponent complex that contains α- and β-chains, CD3 (which is comprised of either ε/δ or ε/γ dimers), in some cases a ζ-chain homo-dimer, as well as a number of accessory proteins (Fu et al., 2014). T-cells can form cell-cell interactions with other lymphocytes, with antigen-presenting cells, and with cells that will be targeted for death. To prevent bystander effects, the proper function of T-cells depends on the secretion of mediators—be they cytokines or cytotoxic effector granules—in a directed and localized fashion. This feat is accomplished by the formation of an “immunological synapse,” which is a specialized and compartmentalized zone of interaction between a lymphocyte and its target cell (Griffiths et al., 2010; Angus and Griffiths, 2013) (Figure 6). Immunological synapses have several important functions including regulation of lymphocyte activation, transfer of peptide-MHC complexes from the antigen-presenting cell to the lymphocyte, and the vectorial secretion of cytokines (e.g., in the case of T_H_ cells) or lytic granules (e.g., in the case of cytotoxic T-cells).

In some regard, the T-cell and its target cell are forming a plasma membrane-plasma membrane MCS, which promotes the transfer of information between the two cells. The synapse is comprised of two major zones: the central supramolecular activation complex (cSMAC), which is comprised of the TCR, its co-receptors, and its associated signaling proteins, and the peripheral SMAC (pSMAC), which is made up of proteins that form an adhesion complex between the interacting cells (Griffiths et al., 2010) (Figure 6). Examples of the latter include the integrin “leukocyte function-associated antigen-1” (LFA-1) on the T-cell and the “intracellular adhesion molecule” ICAM1 on the target cell. Of relevance to this essay is the finding that the *de novo* formation and the function of the immunological synapse are dependent on exocytosis and endocytosis.

The first step in synapse formation is interaction between the T-cell and its target cell, which occurs between the TCR and the peptide-MHC complex on the target cell (Griffiths et al., 2010). This interaction stimulates TCR-dependent signaling, which depends on the action of the Lck, Fyn, and ZAP-70 kinases, as well as the “linker of activated T cells” (LAT). The latter interacts with PLCγ, stimulating IP3-receptor-dependent Ca^2+^ release from the ER and activation of several downstream targets including NFAT (see Figure 3). Because T-cell activation can extend for several minutes to hours, store-operated Ca^2+^ entry is a critical component of T-cell function. Activation of the T-cell leads to formation of integrin-dependent cell-cell interactions. In addition, the actin cytoskeleton undergoes a rapid reorganization that results in its loss from the region of the nacent synapse and polymerization at a region of cell cortex surrounding the pSMAC in a region called the dSMAC (Ritter et al., 2013). Strikingly, the centrosome (and its associated centrioles) moves to a position just below the synapse, followed by movement of the Golgi and recycling endosomes to a similar location (Figure 6). The space created between the cells is then sealed off by the pSMAC.

This organization is very similar to the early steps of lumen formation described in epithelial cysts (Bryant et al., 2010; Apodaca et al., 2012). In both cases, there is a requirement for polarity proteins, reorganization of the cytoskeleton, a repositioning of the secretory and endocytic organelles, and establishment of specialized cell-cell junctions that generate a distinct membrane domain (the apical domain in the case of epithelial cells) (Apodaca et al., 2012; Angus and Griffiths, 2013; Ritter et al., 2013). These similarities indicate that the machinery that drives both processes may overlap and should provide a fertile ground for future exploration.

Activation of the TCR complex is regulated by several factors including the assembly of the TCR complex and the strength of the receptor's signal. Prior to assembly, the intracellular subunits of the TCR-CD3 complex undergo constitutive recycling. Moreover, Lck and Lat are expressed in what appear to be distinct membrane populations (Schade and Levine, 2002), and may use discrete trafficking pathways to the cell surface (Soares et al., 2013). However, the nature of these pathways and the regulatory proteins that govern them are poorly understood. Association of Lck with the CD4 or CD8 accessory chains of the TCR appears to require Rab11a-dependent recycling (Gorska et al., 2009). Moreover, Lck is also associated with MAL, and the Lck activator protein Unc119. The former results in the sorting of Lck and LAT to the center of the immunological synapse. Other Rabs implicated in synapse formation include Rab35 and its effector EPI64C (Patino-Lopez et al., 2008), but how, or if, Rab35 interacts with the putative Rab11a cascade has not been described. In polarized epithelial secretion, Rab11a often operates as an upstream partner in a cascade that includes Rab8 and Rab27/Rab3a (Apodaca et al., 2012). Whether a similar system operates in the formation or operation of the immunological synapse has not been directly addressed, although Rab11a and Rab27a were previously implicated in release of cytotoxic granules in cytotoxic T-cells (Stinchcombe et al., 2001; Menager et al., 2007; Menasche et al., 2008). Interestingly, TCRζ transport to the synapse requires the intraflagellar transport machinery (Finetti et al., 2009), which is critical for formation of cilia, but the details of this process are still in their infancy. There are other similarities between cilia formation and the immunological synapse (Griffiths et al., 2010).

An important role for TCR endocytosis continues once the synapse is formed (Griffiths et al., 2010). Once TCRs interact with MHC/peptide at the periphery of the forming synapse, they move centripitally in the central region of the cSMAC. Here, TCR signaling is terminated as a result of phagocytosis and/or receptor-mediated endocytosis. Furthermore, the lymphocyte can be induced to internalize the peptide-MHC complexes (including bits of the target cell cytoplasm) in a process called trogocytosis, a process that depends on TC21 and RhoG (Ahmed et al., 2008; Martinez-Martin et al., 2011). In contrast, the signaling molecules involved in the initial activation of the T-cell (e.g., Zap70, LAT) appear to be excluded from the TCR microclusters and their signal diminishes after initial delivery to the synapse (Balagopalan et al., 2007), possibly a result of internalization. Indeed LAT may be internalized in a Cbl- and ubiquitin-dependent reaction (Balagopalan et al., 2007). Because the synapse can exist on a minute to hour timescale, it is important to have a mechanism to recruit bystander TCRs (those not engaged with peptide/MHC) to the synapse. One mechanism to promote this recruitment is via β-arrestin1, a well-known adaptor protein that is critical for clathrin-dependent internalization of G-protein coupled receptors. Upon engagement with peptide/MHC, those TCRs in the synapse trigger a PKC-dependent phosphorylation of β-arrestin1 at Ser163, stimulating β-arrestin1 binding to non-phosphorylated, inactive bystander TCRs (Fernandez-Arenas et al., 2014) (Figure 6). Endocytosis of the latter ultimately results in their relocalization to the immunological synapse, where T-cell signaling can be sustained. The machinery that specifies their transcytosis from the free surface to the synaptic membrane is largely unknown, but dependent on β-arrestin1.

An interesting question is whether all membrane traffic is directed toward the immunological synapse of “activated” T-cells. In the case of T_H_ cells, it appears that some cytokines are released in a directed fashion, whereas others are not (Huse et al., 2006; Bertrand et al., 2010). On its face, this indicates that upon activation, T-cells must either use their existing machinery in novel ways, or perhaps synthesize new machinery to ensure polarized secretion. The nature of the polarized secretory machinery is mostly unknown, but as noted above, work in polarized epithelial cells can provide a large number of sorting and regulatory proteins as potential targets (Apodaca et al., 2012). Finally, it is possible that there are other novel mechanisms of secretion that may ensure polarized delivery of cargo. For example, in activated T-cells Golgi stacks appear to abut next to the plasma membrane, perhaps indicating the formation of an MCSs with the synaptic membrane domain (Stinchcombe et al., 2006). While the nature of this interaction and its role in secretion remains to be explored, it indicates that there are numerous things to be learned about how specialized membrane domains are created, maintained, and how they perform special functions. Furthermore, this information is likely to be useful to other cell types including the osteoclast, a bone-degrading cell that forms a synapse-like structure called the “ruffled border” when in contact with the bone matrix (Stenbeck and Coxon, 2014).

## Final words

The important take home point of this essay is that there are many new and exciting areas of research that are in their infancy and will require years to understand. Moreover, there are other exciting areas of research that we have not discussed, but explore fundamental aspects of membrane traffic in normal and disease states. A few of these include: (1) understanding how membrane curvature is regulated to form coated vesicles or membrane tubules (Bechler et al., 2012; Ha et al., 2012); (2) elucidating the global coordination of lipid metabolism, membrane biogenesis, and organelle homeostasis (Henry et al., 2014); (3) manipulating membrane trafficking pathways (e.g., via interventions in phosphoinositide signaling) to control tumorigenesis and intracellular pathogens (Balla, 2013); and (4) elucidating and manipulating the membrane trafficking pathways of proteins that contribute to pathologies including the Alzheimer's disease β-amyloid precursor protein and others leading to neurodegenerative disorders (Zhang et al., 2012). A final Grand Challenge, perhaps the grandest of all, is to elucidate the evolutionary origins of eukaryotic endomembrane systems, i.e., the secretory and endocytic pathways. Ironically, and in spite of the tremendous advances in our understanding of secretion and endocytosis, little is known about how cells evolved to establish these membrane systems (Dacks and Field, 2007; Jekely, 2007). Although much can be inferred from comparative genomics, our understanding of the evolutionary mechanisms by which eukaryotic endomembrane systems developed is rudimentary at best. While the field of membrane traffic is well-established, there is much that remains to be accomplished in the next decade. We hope that this article will inspire new, and established, investigators to pursue these challenging areas of investigation.

### Conflict of interest statement

The authors declare that the research was conducted in the absence of any commercial or financial relationships that could be construed as a potential conflict of interest.
